# Media, messages, and medication: strategies to reconcile what patients hear, what they want, and what they need from medications

**DOI:** 10.1186/1472-6947-13-S3-S5

**Published:** 2013-12-06

**Authors:** Richard L Kravitz, Robert A Bell

**Affiliations:** 1 Department of Internal Medicine and Center for Healthcare Policy and Research, University of California, Davis, Sacramento, CA - 95817, USA; 2 Department of Communication, University of California, Davis, Sacramento, CA - 95817, USA; 3 Department of Public Health Sciences, University of California, Davis, Sacramento, CA -95817, USA; 4 Center for Healthcare Policy and Research, University of California, Davis, Sacramento, CA -95817, USA

## Abstract

**Background:**

Over the past 30 years, patients’ options for accessing information about prescription drugs have expanded dramatically. In this narrative review, we address four questions: (1) What information sources are patients exposed to, and are they paying attention? (2) Is the information they hear credible and accurate? (3) When patients ask for a prescription, what do they really want and need? Finally, (4) How can physicians reconcile what patients hear, want, and need?

**Analysis:**

A critical synthesis of the literature is reported. Observations indicate that the public is generally aware of and attends to a growing body of health information resources, including traditional news media, advertising, and social networking. However, lay audiences often have no reliable way to assess the accuracy of health information found in the media, on the Internet, or in direct-to-consumer advertising. This inability to assess the information can lead to decision paralysis, with patients questioning what is known, what is knowable, and what their physicians know. Many patients have specific expectations for the care they wish to receive and have little difficulty making those expectations known. However, there are hazards in assuming that patients’ expressed desires are direct reflections of their underlying wants or needs. In trying to reconcile patients’ wants and needs for information about prescription medicines, a combination of policy and clinical initiatives may offer greater promise than either approach alone.

**Conclusions:**

Patients are bombarded by information about medicines. The problem is not a lack of information; rather, it is knowing what information to trust. Making sure patients get the medications they need and are prepared to take them safely requires a combination of policy and clinical interventions.

## Background

### Case presentation

*John Doe is a 57-year-old man who has gained 30 pounds over the past 10 years and recently noticed some numbness in his toes. He seeks advice from his family physician. The doctor orders some tests; the results are consistent with adult-onset diabetes. At a follow-up appointment a week later*, *the doctor recommends that Mr. Doe lose weight and writes a prescription for metformin*, *widely considered to be an effective first-line oral hypoglycemic. Mr. Doe fills the prescription and returns home to consider the implications of his diagnosis.*

If this were 1975, the story might end there. Patients interested in learning more about a newly diagnosed condition would talk with family and friends. Some might check out a newspaper, magazine, or tabloid or visit the local bookstore or the library. The occasional, highly motivated patient might dial up the health information line at a local hospital or seek borrowing privileges from the nearest academic medical center library. But since this is 2013, Mr. Doe has more options, including the Internet, social media, and direct-to-consumer advertising (DTCA) of prescription drugs and other medical interventions.

*After receiving the prescription for metformin*, *Mr. Doe does another search on diabetes treatments and comes across an online ad for Januvia^®^* (*sitagliptin*)*. On reading the ad*, *Mr. Doe concludes that Januvia has some real advantages. He even starts to wonder why his doctor failed to prescribe the very best medicine in the first place. Maybe she isn’t keeping up*, *or maybe she’s just trying to keep costs down. Mr. Doe picks up the phone and makes another appointment to see her. He resolves to ask for Januvia in the strongest possible terms.*

This case raises several questions about the health informational environment influencing patients’ attitudes toward and use of prescription drugs. We focus on the following four questions: (1) What information sources are patients exposed to, and are they paying attention? (2) Is the information they hear credible and accurate? (3) When patients ask for a prescription, what do they really want and need? Finally, (4) how can physicians reconcile what patients hear, want, and need?

## Analysis

This analysis takes the form of a critical, integrative synthesis of research from the fields of medicine, marketing, public health, and health communication.

## What sources of information about prescription drugs are available to patients, and are they paying attention?

Health information sources can be classified in various ways. For example, Delorme et al. refer to interpersonal, advertising, and mediated sources [1]. Interpersonal sources include family, friends, and health professionals. Advertising refers to overtly commercial messaging such as DTCA of prescription drugs or drug-company Websites [2-4]. Mediated sources include print and broadcast news, entertainment programming, and the Internet. Social media such as Facebook, Twitter, and online support groups are sources of health information that occupy an increasingly important middle ground between face-to-face interpersonal and traditional media [5,6].

The health information environment is changing rapidly. Some major newspapers are successfully shifting their focus from print to the Internet, while others are struggling to survive [7]. As newspapers contract, they tend to lay off specialized health reporters trained to sort through complex stories and place them in context [8]. The same trends are evident in broadcast news, where the number of health stories is increasing but the average airtime per story is decreasing [9]. Meanwhile, the Internet and DTCA offer abundant information, but with little assurance against subtle bias, commercialism, or deception. In short, while the public has greater access to health information than ever, they are increasingly vulnerable to potentially misleading information.

Although people differ in their health consciousness [10], evidence suggests that many are aware of and responsive to the health information environment. A Kaiser Family Foundation survey of 42,000 Americans covering the period 1996–2002 showed that, overall, 4 in 10 adults follow health news stories closely [11]. People appear to pay the most attention to stories that are alarmist (“if it bleeds it leads” [12]) or are personally relevant [13-15]. This may explain why, in the Kaiser survey, public health hazards and common diseases like cancer and Alzheimer’s disease drew more attention from the public than specific prescription drugs, which directly affect a small proportion of the population (i.e., those who are taking the drugs or know someone who is). Nevertheless, certain high-profile drug stories have had considerable impact. For example, publication and subsequent media coverage of the Heart and Estrogen/Progestin Replacement Study and the Women’s Health Initiative were associated with significantly reduced use of hormone replacement therapy in postmenopausal women; at least part of the prescriber effect was likely mediated through consumers [16].

The Internet has revolutionized health information seeking in particular. Use of the Internet for health information seeking is widespread and growing [4,5,17,18], and such use often both precedes and follows medical visits [19,20]. However, uneven uptake of new technologies raises equity concerns. For example, a Pew report suggests that adults with chronic diseases (i.e., those presumably most in need of the information) are *less* likely to go online for health information than adults reporting no chronic diseases (62% vs. 81%) [21]. While this might be explained in part by age, other studies have shown disparities in Internet health information seeking between rich and poor, whites and minorities, and young and old [22-25]. However, the socioeconomic digital divide is shrinking, and some studies suggest that use of mobile Internet platforms is greater among minorities than among whites [26].

DTCA of medications has occurred since at least the 18th century [27], but DTCA of prescription drugs accelerated in 1997 when the U.S. Food and Drug Administration (FDA) relaxed its interpretation of standards limiting broadcast advertising [28]. Awareness of DTCA among consumers is very high [3,29-31]. Survey research and analyses of insurance claims suggest that consumers notice prescription drug advertisements and act on them by making additional physician visits, requesting prescriptions, and occasionally registering dissatisfaction when requested drugs are not provided [2,32-35]. There is also evidence that drug manufacturers have successfully expanded the potential market for their products by not only encouraging patients with chronic conditions to stay on (adhere to) their medications but also through “disease mongering” [36,37]; medicalizing common symptoms such as mild depression [38] and leg movements during sleep [39], and legitimizing contested illnesses [40].

In summary, consumers are swimming in a sea of health-related information, but paradoxically they may still feel unprepared to participate in health-care decisions [41]. Part of the problem is the paucity of comparative effectiveness data, a problem that may never be fully resolved [42]. Another aspect of the problem is the relative lack of patient-friendly materials addressing the full spectrum of risks and benefits of treatment. Initiatives launched by organizations like the American Medical Association, the Cochrane Collaboration, the Society of General Internal Medicine, and the Agency for Healthcare Research and Quality are beginning to assemble patient-friendly, integrated information for decision making, but a comprehensive solution will likely require government action.

## Can patients trust what they see and hear?

The question of whether patients *can* trust what they pick up from the health information environment is inextricably bound to the question of whether they *should* trust the information. In this brief analysis we will distinguish *credibility* (whether patients subjectively trust the information they hear about medications) from *accuracy* (the degree to which the information *merits* trust on the basis of medical evidence).

Consumer judgments regarding the credibility of health information depend on characteristics of both the *source* and the *message*. Evaluations of source credibility derive from a person’s judgment of the information source’s *expertise* (how much the person or organization is thought to know) and *trustworthiness* (the degree to which the source is believed to say what he or she believes to be true) [43]. Such evaluations may depend on personal knowledge of the source (e.g., “homophily” or friendship, membership in the same social network [44]), or on cues that signal reputational quality (e.g., a report published by a well-known national organization such as the Centers for Disease Control and Prevention or the American Cancer Society). On the Internet, consumers appear to take account of content comprehensiveness and Website complexity [45]. However, several observational studies suggest that when evaluating online health information, consumers have difficulty evaluating source credibility [46].

In terms of message characteristics, studies have confirmed the importance of information quality, personalization, impartiality, and richness of message features (such as references and testimonials) [47,48]. However, there may be critical interactions between patient characteristics (e.g., their health numeracy skills) and evaluation of message quality [49]; patients with poor quantitative skills may become frustrated by too much numerical information, whereas those with better skills and greater sophistication may desire and expect more numerical detail [50]. In fact, health literacy may determine to a great extent whether patients understand medication-related materials, including FDA warning labels [51]. Patients unable to attain a minimal threshold of understanding cannot be expected to extract benefit from such materials, no matter how expertly written.

Regardless of what consumers may think, inaccurate health information is common, even when reported by well-known news outlets and reputable Websites [52]. Four phenomena threaten the trustworthiness of prescription drug information as disseminated to the public. First, the complex alliance of “Big Pharma,” government, academic institutions, and medical journals is riddled by problems including seeding trials (marketing campaigns aimed at medical opinion leaders, disguised as clinical trials), ghostwriting, and selective publication [53]. Second, reporters frequently omit information about drug company sponsorship of clinical trials and tend to collude unintentionally with drug manufacturers by using trade names rather than generic names. Reporting about results presented at scientific meetings tends to be particularly intemperate [54,55]. Third, reporters often fail to supply quantitative information, provide only relative measures of impact, or misconstrue the meaning of statistical significance [56]. Relative measures (e.g., “a 20% reduction in risk of death”) can be misleading if not accompanied by base rates or absolute difference measures (e.g., “a reduction in risk of death from 1% to 0.8%”). Similar errors of omission have been noted across health Internet sites [17]. Fourth, as seen with the rotavirus vaccine, there can be a tendency to overplay benefits early in the life cycle of a product, followed by sudden condemnation at the first sign of unanticipated adverse effects [57]. The cacophonous and not wholly trustworthy information environment is without doubt one reason patients consistently report relying on physicians and pharmacists for advice on medications [58]. A recent survey of patients suggests that those who have relied upon medication information from the media, brochures, family, and friends are less likely to adhere to their treatment plans [59].

In contrast to news stories, the commercial origins of DTCA are usually obvious. Nevertheless, consumers often find these medication ads compelling, because the ads engage their audience by appealing to emotions [60,61] and by highlighting effectiveness, convenience, symptom control, and innovativeness but not cost [62]. However, the medication ads frequently do not address critical issues, such as how to manage chronic conditions, and they are formatted in ways that give low-literacy consumers difficulty [63]. Quantitative data on effectiveness are scarce [64]. When asked directly, many patients report being skeptical of DTCA claims [65]. They have good reason to be; the accuracy of information provided about some therapeutic categories such as chemotherapy is highly questionable [66]. Nonetheless, DTCA clearly motivates patients to ask questions [67], request prescriptions [68], and even seek drugs directly off the Internet; one in five Americans report having asked their doctor for a prescription based on a medication ad [69]. In addition, even skeptical persons may be susceptible to DTCA over time, as the advertising claims become disassociated with discredited sources (the “sleeper effect”) [70,71].

In summary, the public has no reliable way to assess the accuracy of health information found in the print and broadcast media, on the Internet, or in DTCA. This can lead to decision paralysis, with many patients questioning what is known and knowable and with a few even questioning what their physicians know.

## What do patients really want and need?

Most patients seek formal medical care with a goal in mind; they hope to gain answers to questions, relief of symptoms, reassurance, or a specific intervention (e.g., a test, a referral, or a prescription). When queried directly, patients often report having highly specific expectations for care. For example, one study performed in a general internal medicine group practice showed that a majority of patients wanted examinations of their head and neck, lungs, heart, and abdomen in addition to blood testing; but, an even larger group wished for counseling about prognosis and discussion of the patient’s own ideas about management [72]. A follow-up study of audio recordings of 559 ambulatory visits showed that approximately one-quarter of patients in primary care and cardiology made observable requests for tests (8%), new medications (11%), or specialty referrals (5%). Patients’ requests for specialty consultations or medications independently predicted whether they received those services [73]. When patients’ expectations are not fulfilled, they tend to experience less improvement in health, have weaker intentions to adhere to treatment, and report lower satisfaction with the visit [74].

Do patients’ requests reflect their true underlying desires? Two lines of evidence suggest not. First, in qualitative studies and survey research, patients consistently emphasize the importance of interpersonal care, such as the need for the physician to listen and provide information [75]. In contrast, studies using direct observation (audiotaping or videotaping) tend to document requests for technical care (e.g., a prescription) or clinical information (“what are the side effects of this medicine?”) [73,76]. Evidently, and unsurprisingly, it is more difficult to ask for a supportive relationship than for a computerized tomography scan of the chest. In addition, even if patients recognize their own needs, they do not always voice them [77]. Second, in a follow-back interview study of 125 patients with unmet expectations for care, many patients assigned special significance to the services they expected or requested. For example, 50% of patients ascribed the genesis of their expectations for physician interventions to perceived vulnerability (e.g., “My father had a heart attack at 40, and I’m 42—he should at least listen to my heart!”) and 42% to past experience with a similar illness (“My other doctor always gave me antibiotics for this sore throat, and it got better right away.”).

If patients cannot always get what they want, can they at least get what they need? This begs the question of how to define need, something that has frequently vexed experts [78,79]. Without returning to first principles, we suggest that patients need at least three things from their doctors: valid evidence, clinical discernment, and a healing relationship. Accurate information is particularly important to highly engaged patients in noncritical clinical situations, but even patients with serious illnesses like cancer or amyotrophic lateral sclerosis value shared decision making (up to one-third in some studies) [80]. Despite the ready accessibility of information on medications, patients still need help from their clinicians to identify high-quality evidence and interpret it in light of their own clinical circumstances. And research has consistently upheld the importance to patients of clinicians who listen carefully, communicate effectively, and convey compassion and concern [81,82].

There are hazards in assuming that patients’ expressed desires are direct reflections of their underlying wants or needs. Simply acceding to patients’ requests for medications, for example, may have a number of potentially deleterious consequences. For medicines with known serious side effects or that are relatively new to the market, providing an equivocally indicated prescription places the patient at risk for adverse events without countervailing benefit [83]. Some medicines may require periodic monitoring with laboratory or imaging studies, which themselves can trigger a “cascade effect” of false-positive results that are followed by ever more invasive testing [84]. Furthermore, focusing on the literal wording of a request may distract attention from more significant underlying issues (e.g., fear of disease progression, disability, disfigurement, or death), leading to a cycle of anxiety and amplification of symptoms [85].

## How can conflicts between patients’ wants and needs be reconciled?

In our increasingly chaotic health information environment, satisfying patients’ wants while ensuring that their medication needs are met is a difficult challenge. We suggest a combination of policy and individual-level solutions.

Policy solutions should include interventions aimed at improving the quality of the prescription drug information available to consumers, improving the clarity of consumer-directed signals that indicate reputational quality and trustworthiness, and limiting the influence of commercial bias in the production and dissemination of information about medicines. The quality of prescription drug information could be improved through a requirement that consumer drug information include details on absolute risks and benefits of therapy [86,87]; development of standards and training for news reporting of health-related stories (possibly through organizations such as http://healthjournalism.org) [88,89]; and arguably, formation of an independent agency, funded in part by the pharmaceutical industry, that would produce unbiased consumer materials [90]. These efforts should take full advantage of advances in our understanding of how people perceive risks and how risk information can best be conveyed [91]. Better reputational signaling (at least on the Internet) could be achieved through broader uptake of standards such as those promulgated by the Health on the Internet Foundation, which is a nongovernmental association (http://www.hon.ch). The organization's HONcode guidelines are now used by over 7300 Websites in more than 100 nations. Commercial bias could also be checked through clinical trial registration; more transparency in reporting conflicts of interest; and, arguably, a 2-year moratorium on DTCA of new drugs [90].

These policy prescriptions would help, but their adoption and implementation will take time. Meanwhile, clinicians see large numbers of patients everyday who are caught up in a media and advertising frenzy that manufactures new afflictions and urges “a pill for every ill” [92]. Prescription requests are common in office practice, and physicians need to know how to respond. Our recommendation is that the response be guided not just by the plain language of the request, but by the physician’s interpretation of the patient’s reasons for asking. While implementing some of these recommendations may require the investment of additional time in the beginning, they will increase efficiency in the long run [93].

When patients talk about the genesis of their expectations for prescription medicines, they tend to emphasize personal vulnerability and past experiences more than the influence of specific news stories or DTCA [94]. A request for medication is often a veiled request for understanding, empathy, concern, reassurance, or solidarity. The prescription is a way of saying: “I take your concerns seriously, and I am going to use my special powers as a physician to address them.” Therefore, the physician’s first step should be to clarify what patients really want. Instead of interrupting within the first 18 to 21 seconds [95,96], physicians should stop talking (or typing on the computer) and allow patients to finish their opening statement [97], which rarely takes more than 3 minutes. An occasional “uh huh,” “something else?” or “anything else?” will facilitate disclosure of the patient’s full agenda. Once the agenda is clear, the stage is set for a more productive clinical negotiation [98].

Second, physicians should resist the temptation to provide encyclopedic quantities of information unless it is clear that this is what the patient wants. It is easy to assume that when patients ask questions or request interventions, they want specific facts or action; however, this may or may not be the case. Patients will be more comfortable with the physician’s response if they are first assured that their question was understood. Therefore, when a patient asks for prescription medicine, the physician should respond with more questions: “How did you hear about that medicine?” “How were you hoping it would help you?” “Are you having any problems with the medicine you’re already on?” When the physician understands the underpinnings of the request, it is easier to formulate a response that is both clinically appropriate and mutually satisfying [99]. The physician may also consider “prescribing” visits to Websites that offer unbiased drug information, such as the U.S. National Library of Medicine's MedlinePlus^®^ or http://ConsumerReports.org.

Third, physicians should be alert to patients’ emotional clues. Work by Levinson et al. suggests that physicians frequently miss opportunities to respond to patient emotions such as frustration, anger, and despair [100]. These cues are often conveyed nonverbally [101-103]. By acknowledging patients’ distress, providing encouragement, and offering support, physicians can help patients overcome obstacles to effective self-care [100,104].

Fourth, physicians need to be aware of their own emotions [105]. Patients’ requests for prescriptions can provoke a spectrum of negative emotions in physicians, including defensiveness, anger, anxiety, sadness, and guilt. Awareness of these emotions can mitigate their destructive consequences.

Finally, in negotiating with patients who seem to want medicines that are not deemed in their best interest, physicians have to give something to get something. Three strategies have been widely adopted. The “substitution strategy” reframes the diagnosis (“The most likely explanation for your fatigue is stress from work.”) or offers an alternative therapeutic approach (“What we need to do is get you on a regular exercise program.”) [99]. The “contingency strategy” involves offering a therapeutic trial of the physician’s preferred therapy (or watchful waiting) with the option to switch to the patient’s preferred treatment after an agreed-on period of time without improvement (“Here’s an antibiotic prescription, but don’t fill it if you begin to see improvement before Wednesday.”) [106]. In a closely related approach (the “availability strategy”), the physician makes himself or herself available to the patient as a bulwark against anxiety (“I’ll call you in the morning on Thursday to make sure you’re doing better.”). In a trial involving standardized patients portraying depression, use of these strategies for “getting to no” were associated with better subjective evaluations of the physician when compared with simply rejecting or ignoring the request [99].

## Conclusions

Returning to the questions posed at the beginning of this article:

• Patients are bombarded by information about medicines. The problem is not a lack of information, it is knowing what to trust.

• The quality of prescription drug information available to consumers is variable, and consumers have difficulty discriminating between reliable and less reliable sources of information.

• When patients ask for medications, they may be seeking something else entirely. It is the clinician’s job to sort this out. This need not take much time.

• Making sure patients get the medications they need and are prepared to take them safely requires a combination of policy and clinical interventions that also give due consideration to nonpharmacologic treatments.

## List of abbreviations used

DTCA: direct-to-consumers advertising; FDA: U.S. Food and Drug Administration

## Competing interests

Drs Kravitz and Bell declare that they have no competing interests.

## Authors’ contributions

Drs RLK and RAB have worked together for almost two decades and regularly share ideas, including many that appear in this manuscript. Dr. RLK wrote the first draft; Dr. RAB edited the manuscript extensively and added many references. Both authors take responsibility for the final product.

## Declarations and disclaimer

The Eisenberg Conference Series 2012, *Supporting Informed Decision Making When Clinical Evidence and Conventional Wisdom Collide*, was conducted in Rockville, Maryland, by the John M. Eisenberg Center for Clinical Decisions and Communications Science, Baylor College of Medicine, Houston, Texas, for the Agency for Healthcare Research and Quality under Contract No. HHSA 290-2008-10015-C. Publication costs for this supplement were funded by this contract. The author of this article is responsible for its content. No statement may be construed as the official position of the Agency for Healthcare Research and Quality and of the U.S. Department of Health and Human Services.

This article has been published as part of *BMC Medical Informatics and Decision Making* Volume 13 Supplement 3, 2013: Articles from the Eisenberg Center Conference Series 2012: Supporting informed decision making when clinical evidence and conventional wisdom collide. The full contents of the supplement are available online at http://www.biomedcentral.com/bmcmedinformdecismak/supplements/13/S3.
